# Previous Musical Experience and Cortical Thickness Relate to the Beneficial Effect of Motor Synchronization on Auditory Function

**DOI:** 10.3389/fnins.2019.01042

**Published:** 2019-09-27

**Authors:** Natascha Merten, Johanna Kramme, Monique M. B. Breteler, Sibylle C. Herholz

**Affiliations:** ^1^Population Health Sciences, German Center for Neurodegenerative Diseases, Bonn, Germany; ^2^Institute for Medical Biometry, Informatics and Epidemiology (IMBIE), Faculty of Medicine, University of Bonn, Bonn, Germany

**Keywords:** frontal pole, frontopolar cortex, Heschl’s gyrus, melody, rhythmic finger tapping

## Abstract

Auditory processing can be enhanced by motor system activity. During auditory-motor synchronization, motor activity guides auditory attention and thus facilitates auditory processing through active sensing. Previous research on enhanced auditory processing through motor synchronization has been limited to easy tasks with simple stimulus material. Further, the mechanisms and brain regions underlying this synchronization are unclear. We investigated the effect of motor synchronization on auditory processing with naturalistic, musical auditory material in a discrimination task. We further assessed how previous musical training and cortical thickness of specific brain regions relate to different aspects of auditory-motor synchronization. We conducted an auditory-motor experiment in 139 adults. The task involved melody discrimination and beat tapping synchronization. Additionally, 68 participants underwent structural MRI. We found that individuals with better auditory-motor synchronization accuracy showed improved melody discrimination, and that melody discrimination was better in trials with higher tapping accuracy. However, melody discrimination was worse in the tapping than in the listening only condition. Longer previous musical training and thicker Heschl’s gyri were associated with better melody discrimination and better tapping synchrony. *Post hoc* analyses furthermore pointed to a possible moderating role of frontal regions. Our results suggest that motor synchronization can enhance auditory discrimination abilities through active sensing, but that this beneficial effect can be counteracted by dual-task inference when the two tasks are too challenging. Moreover, prior experience and structural brain differences influence the extent to which an individual can benefit from motor synchronization in complex listening. This could inform future research directed at development of personalized training programs for hearing ability.

## Introduction

Auditory functions are essential for everyday interactions. Strategies that improve impaired hearing abilities therefore have the potential to improve quality of life. Two factors that are closely related have the potential to positively influence hearing abilities: musical training and auditory-motor synchronization. Previous studies have shown that life-long musicianship and early life musical training have not only a positive effect on sensory-motor (Penhune and Steele, 2012) but also on auditory processing (Herholz and Zatorre, 2012). Even relatively short-term musical training has been associated with changes in brain morphology and physiology subserving auditory processing (Herholz and Zatorre, 2012). The strong auditory-motor interactions that are specific to music are one of the driving forces for neuronal plasticity (Zatorre et al., 2007; Herholz and Zatorre, 2012).

Several studies have begun to explore whether auditory-motor synchronization can enhance auditory perception. These studies investigated different aspects of non-verbal auditory processing, namely rhythmic and pitch processing. An immediate effect of motor synchronization on rhythm and timing perception has been shown (Su and Pöppel, 2012; Manning and Schutz, 2013; Schmidt-Kassow et al., 2013). Furthermore, Morillon et al. (2014) showed an immediate top–down influence of finger beat tapping on pitch discrimination of non-melodic tone sequences. The proposed mechanism for this is an interaction between auditory, motor, and attention systems that enhances the processing of auditory information (Morillon et al., 2014; Mottonen et al., 2014). The rhythmic motor routine supposedly sharpens sensory representations and facilitates perception of relevant items while it suppresses irrelevant items, enacting auditory ‘active sensing.’ More specifically, the bottom–up information of the auditory and motor activity may build up a temporal prediction of the sensory event. Additionally, top–down attentional control mechanisms align the rhythmic fluctuations in sensory gain with the rhythm of the incoming sensory input in order to enhance processing (Morillon et al., 2014). This suggestion is in line with research on the complex interaction of auditory, motor, and top-down cognitive processes, such as attention, in musical performance in general (Zatorre et al., 2007).

It is not entirely clear which brain regions are involved in this type of auditory-motor synchronization. Cerebellum, basal ganglia, and supplementary motor area (Zatorre et al., 2007) are involved in timing of movement. Basal ganglia, superior temporal gyrus, premotor cortex, and ventrolateral prefrontal cortex are relevant for beat tapping (Kung et al., 2013). A major auditory processing region is the Heschl’s gyrus which is relevant for pitch perception (Zatorre, 1988; Patterson et al., 2002; Norman-Haignere et al., 2013) as well as retention of rhythmic patterns (Penhune et al., 1999).

In the present study, we aimed to assess a beneficial effect of motor synchronization on auditory discrimination performance with naturalistic and meaningful auditory material, namely short melodies in the Western tonal system. Based on the active sensing theory, we hypothesized that better motor synchronization is related to better melody discrimination and that there is a beneficial effect of motor synchronization compared to listening only. Furthermore, we aimed to test to what extent musical training and brain anatomy of auditory (Heschl’s gyrus) and premotor areas affect both motor synchronization accuracy and melody discrimination. In the course of the analysis our results prompted further exploratory *post hoc* analyses regarding individual differences in motor synchronization abilities and brain anatomy of frontal brain areas.

## Materials and Methods

### Participants

The experiment was performed in 148 participants of the pilot phase of the Rhineland Study. The Rhineland Study is a recently started prospective cohort study. Participants of the pilot study were recruited via newspaper advertisements of local student services. We did not recruit them according to their musical training experience. All participants were healthy, fluent speakers of German, and at least 18 years of age. Approximately half of the participants were invited to an MRI scan. This invitation to an MRI scan was unrelated to any of the criteria of the experiment.

This study was carried out in accordance with the recommendations of the International Council for Harmonisation (ICH) Good Clinical Practice (GCP) standards (ICH-GCP) with written informed consent from all subjects. All subjects gave written informed consent in accordance with the Declaration of Helsinki. The protocol was approved by the ethics committee of the University of Bonn, Medical Faculty.

### Study Procedure

For the present study, participants performed a melody experiment and answered a short questionnaire on musical background. Moreover, MRI scans were available for 72 of those participants.

### Melody Experiment

In the melody experiment the main task was a simple melody condition where participants had to decide by button press whether or not two melodies in a row were the same (Foster and Zatorre, 2010a, b). In half of the trials the pitch of a single note was changed by up to ±5 semitones (median of 2 semitones). This change maintained the key and the melodic contour (the order of upward and downward pitch movement in a melody without regard to magnitude) of the melody. The melody stimuli were 5–13 notes per melody, low pass-filtered harmonic tones with pitches between C4 and E6. All notes were 320 ms in duration, equivalent to eighth notes at a tempo of 93.75 beats per minute, that is, all melodies had an isochronous rhythm. Varying the number of notes among trials ensured a sufficient range of difficulty and sensitivity across the full range of musical experience in our sample (Foster and Zatorre, 2010a,b). All stimuli were presented at a comfortable hearing level.

**FIGURE 1:**
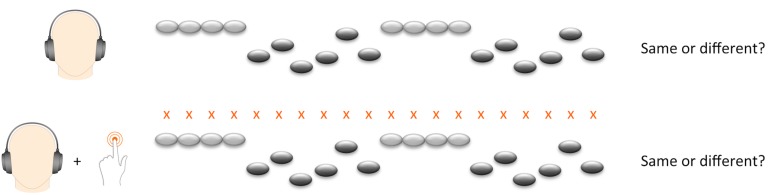
Melody experiment paradigm.

We presented the melody task in two experimental conditions (Figure 1). In half of the trials the participant merely listened to the melodies, whereas in the other half of the trials they tapped along to the beat of the melody with the left index finger. Since neuronal activity related to pitch discrimination is right-lateralized (Zatorre and Belin, 2001), and since we wanted to maximize the potential influence of motor synchronization, we asked participants to tap with their left index finger in order to enhance neuronal activity in the right-hemispheric network. Participants were required to tap on a touch-sensitive tablet computer screen placed horizontally on the table within comfortable reach of their hand.

In order to introduce participants to the beat before the start of the first melody, all trials started with four beats presented in a wooden sticks sound. The beat also continued during the interval between the first and second melody, bridging the two melodies with four beats. In the tapping condition, participants were instructed to start tapping on the first beat and to continue throughout the whole trial, including the interval between the two melodies. Before the experiment, participants practiced tapping to the beat for 30 s and performed four exercise trials of the tapping condition. We divided 72 trials in four blocks, with tapping and listening conditions alternating twice. We used four different versions of trial orders. Versions and order of conditions were counterbalanced across participants. Total task duration was approximately 18 min. The task was conducted on a Samsung Galaxy note 10.1 2014 edition with Sennheiser HD 201 earphones.

Melody discrimination performance (MDP) was computed as percent correct answers on melody discrimination task. We computed this sum score across all trials as well as separate scores for the tapping and listening conditions. Additionally, we computed a score of tapping performance for each melody trial, the sensorimotor simultaneity index (SSI). To compute the SSI, we first established a theoretical reference beat corresponding to occurrences of the target taps each 320 ms along the sequence of tones of the trial. We corrected for a possible delay in the recording of the motor acts by extracting the motor-tracking sequence and aligning it to the theoretical reference beat number one so as to minimize the trial-averaged delay between the two sequences (Morillon et al., 2014). On each trial, we excluded the first four beats before the start of the melody from the estimation of the SSI since they served as introductory phase to find the beat. SSI was defined as the mean absolute temporal distance between target taps and actual taps and describes the ability to closely match the tone onset. A higher score refers to worse tapping ability. We used both trial-by-trial SSI scores and a sum score of mean SSI across all tapping trials per participant in the statistical analyses. In order to ensure valid estimation of tapping performance per trial, trials with more than [mean number of taps + three standard deviations] missing taps per melody (equivalent to 37% of taps per melody) were excluded. After this step, if more than 10% of trials per participant were missing, the participant was excluded to ensure valid estimation of tapping performance per participant.

### Questionnaire

The following data were collected using a self-report questionnaire: sex, age, handedness, and history of musical training, which included years of musical training and starting age of musical training. Starting age was missing for one individual who therefore was omitted from analyses including starting age as a variable.

### Magnetic Resonance Image Acquisition and Processing

**FIGURE 2:**
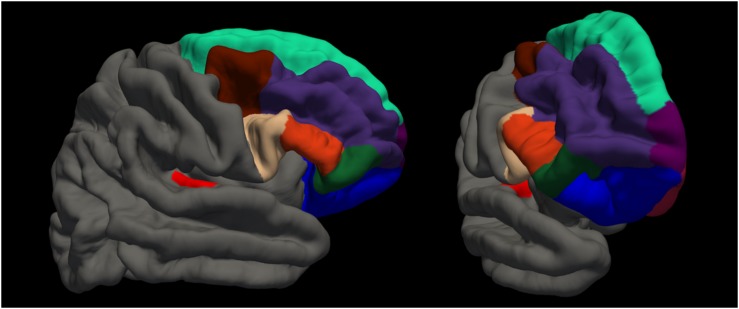
Visualization of regions of interest. red: transverse temporal cortex (Heschl’s gyrus); beige: pars opercularis; orange: pars triangularis; dark green: pars orbitalis; blue: lateral orbitofrontal cortex; brown: caudal middle frontal cortex; dark purple: rostral middle frontal cortex; turquoise: superior frontal cortex; light purple: frontal polar cortex; dark red: medial orbitofrontal cortex.

Magnetic resonance imaging (MRI) sessions were scheduled on the same day as the behavioral examinations. T1 and FLAIR scans were acquired alongside other sequences of the Rhineland Study MRI pilot protocol within a 50 min session on a Siemens 3 Tesla scanner (Siemens, Prisma Magnetom, Erlangen, Germany). Both structural images were acquired with whole brain coverage and an isotropic 1 × 1 × 1mm resolution with an MPRAGE sequence (field of view: 256 × 256 mm, repetition time: 2530 ms, echo time: 2.83 ms, flip angle: 7.0°, acquisition time: 4:57 min) and a FLAIR sequence (field of view: 256 × 256 mm, repetition time: 5000 ms, echo time: 393 ms, flip angle: 120°, acquisition time: 4:42 min). We processed the data according to the recommended surface-based analysis stream for cortical surface segmentation in FreeSurfer Version 5.3 (Dale et al., 1999; Fischl et al., 1999) including registration to the MNI305 atlas, skull stripping and classification of voxels as white and non-white matter. For each hemisphere separately, the algorithm computes surfaces to separate white from gray matter (white surface), and gray matter from CSF (pial surface). FLAIR data were used to improve surface estimates. Finally, cortical thickness estimates were generated from the distance between white and pial surface. Then, based on the Desikan atlas (Desikan et al., 2006), we extracted average cortical thickness values for each individual for the following regions of interest (ROIs): right and left transverse temporal cortex (Heschl’s gyrus), and right superior frontal cortex (including premotor areas, see Figure 2). We further extracted the estimated intracranial volume (Buckner et al., 2004).

### Statistical Analyses

To check the quality of our designed experiment we examined several aspects. Using univariate analyses of variance, we investigated possible performance differences due to the experimental version and performance increase or decrease over the four experimental blocks in MDP due to learning or fatigue, respectively. The reliability of MDP was tested using the formula *r*_Spearman–Brown_ = Pearson correlation of test halves (Eisinga et al., 2013).

For all linear models we winsorized extreme values of SSI (defined as values deviating more than three standard deviations from population mean). We log-transformed highly skewed data on starting age of musical training and *z*-standardized the cortical thickness values of the ROIs. The threshold for statistical significance was set at α < 0.05.

We investigated the effect of motor synchronization on MDP on a trial and on a subject level. Firstly, we investigated the effect of SSI on the probability to give a correct melody discrimination answer on each trial. Here, SSI varies across trials for each participant. We used a generalized linear mixed model (GLMM) to account for repeated measures. We defined SSI per trial as independent variable and correctness (0 versus 1) of the melody discrimination answer of the respective trial as dependent variable. Since the outcome was binary we used a logit link function. We adjusted for random effects of trial and participant and reran the model with additional adjustment for years of musical training and trial length. Secondly, we tested the effects of SSI and of experimental condition (tapping versus listening) on MDP. Here we used average SSI across all tapping trials as index for overall motor synchronization ability that varies across participants. We used a linear mixed effects model (LMEM) with average SSI and the experimental condition (tapping versus listening) as independent variables and MDP as dependent variable. We controlled for the random effect of subject and reran the model with adjustment for years of musical training. We conducted an additional *post hoc* analysis to explore a possible relationship of tapping abilities with the beneficial or detrimental effect of tapping. We ran a linear mixed-effects model at subject level with MDP as dependent variable and main effects of musical training years, experiment condition (tapping versus listening), and the interaction between experiment condition and musical training years as independent variables controlling for random effect of participant.

To examine whether musical training relates to the auditory component or the motor component of auditory-motor synchronization we used linear regression models with years of musical training and starting age of musical training as independent variables and MDP or SSI as dependent variables.

We compared sample characteristics between the total sample and the subset with MRI using chi-squared tests and univariate analyses of variance. In the MRI subsample, we then investigated whether cortical thickness in Heschl’s gyrus or right superior frontal cortex were related to the auditory or motor component of auditory-motor synchronization with linear regression analysis. We used models with Heschl’s gyrus (left and right hemisphere separately) cortical thickness as independent and MDP or SSI as dependent variable. Furthermore, we used a model with right superior frontal cortex cortical thickness as independent and SSI as dependent variable. We adjusted all models for intracranial volume and also reran the analyses with additional adjustment for musical training years. To control for handedness, we assessed whether the observed effects remained consistent in the sub-sample of right-handed individuals.

We ran additional *post hoc* analyses to explore possible relationships of the beneficial or detrimental effect of tapping with cortical thickness in frontal regions. We hypothesized that the harmful effect could be due to dual-task inference and therefore brain regions for attention control might be of relevance. While attention networks are thought to involve a widely distributed network of fronto-parietal regions (Corbetta and Shulman, 2002; Buschman and Miller, 2007), particularly the prefrontal cortex has a major role in top–down attention control mechanisms (Miller and Cohen, 2001). We assessed the following regions in both hemispheres, according to the Desikan atlas (Desikan et al., 2006): lateral orbitofrontal, medial orbitofrontal, pars orbitalis, pars triangularis, pars opercularis, rostral middle frontal, caudal middle frontal, frontal pole (see Figure 2). To explore these relationships, for each region, we first plotted the difference in MDP between the experiment conditions (tapping and listening) as a function of cortical thickness. Then, we used LMEM with main effects of cortical thickness, experiment condition (tapping versus listening) and the interaction between experiment condition and cortical thickness of the respective area as independent variables and MDP as dependent variable. We corrected for intracranial volume, years of musical training, and random effect of participant.

Statistical procedures were performed in Matlab Version R2015b (The Mathworks Inc, 2015) and R Version 1.0.44 (R Core Team, 2015) with packages dplyr (Wickham and Francois, 2015), tidyr (Wickham, 2014), moments (Komsta and Novomestky, 2015), car (Fox and Weisberg, 2011), lmertest (Kuznetsova et al., 2015), lme4 (Bates et al., 2015), and ggplot2 (Wickham, 2009).

## Results

Of the 148 participants who conducted the melody experiment we had to exclude one participant with only 22.2% correct answers in the last block, assuming concentration issues or failure to follow instructions, and further 8 participants because of missing data in SSI. Of the remaining 139 participants MRI data were available for 68 subjects. Table 1 presents the descriptive characteristics of the participants of the total sample and the MRI subsample. Samples did not significantly differ in any of the reported characteristics (*p* > 0.25). For those participants who received musical training (*n* = 88) there was a wide range of years (between 1 and 16) and starting age (4–21) of musical training. On average, participants answered correctly 70.1% (*SD* = 9.5%) of the trials and their taps deviated 32.7 ms (*SD* = 14.9 ms) from tone onset.

**TABLE 1:** Descriptive characteristics of participants of total and MRI subsample.

	**Total sample**	**Subsample with MRI**
*N*	139	68
Age *M*(SD) [years]	25.37 (4.35)	24.69 (4.03)
Sex		
Women *N*(%)	83 (59.71)	42 (61.76)
Men *N*(%)	56 (40.29)	26 (38.24)
Musical training *N*(%)	88 (63.31)	46 (67.65)
Musical training *M*(SD) [years]	4.34 (4.76)	4.63 (4.73)
SA of musical training *M*(SD) [years]	8.43 (3.33)	8.07 (3.15)
Handedness		
Left or both *N*(%)	15 (10.79)	9 (13.24)
Right *N*(%)	124 (89.21)	59 (86.76)
MDP total *M*(SD) [% correct]	70.07 (9.48)	71.26 (8.83)
MDP listening *M*(SD) [% correct]	71.00 (11.00)	72.18 (10.52)
MDP tapping *M*(SD) [% correct]	69.14 (10.64)	70.34 (9.66)
SSI *M*(SD) [ms]	32.69 (14.93)	31.71 (14.07)
Right HG thickness *M*(SD) [mm]	–	2.55 (0.20)
Left HG thickness *M*(SD) [mm]	–	2.50 (0.21)
Left SFC thickness *M*(SD) [mm]	–	2.80 (0.14)
Right FPC thickness *M*(SD) [mm]	–	2.81 (0.24)
Left FPC thickness *M*(SD) [mm]	–	2.81 (0.23)

*SA, starting age; MDP, melody discrimination performance; SSI, sensorimotor simultaneity index; HG, Heschl’s gyrus; SFC, superior frontal cortex; FPC, frontopolar cortex.*

Melody discrimination performance showed an acceptable split-half reliability (*r* = 0.77). There was neither a significant effect of experimental version on MDP nor a significant performance increase or decrease over the four blocks (*p* ≥ 0.14).

### Effect of Tapping Accuracy on Melody Discrimination

In the first part of our analyses we aimed to directly link tapping accuracy with MDP, both at the subject and at the trial level.

#### Subject Level

We found that participants who were able to synchronize more accurately with the auditory stimulus (as measured by their average SSI score) performed better on the melody discrimination task (−0.25% difference per ms; *t* = −4.99; *p* < 0.001). Adjusting for years of musical training slightly decreased this effect (−0.18% difference per ms; *t* = −3.81; *p* < 0.001).

#### Trial Level

We found that more precise tapping (SSI) in a specific trial increased the probability of a correct answer in the melody discrimination task in the same trial (−0.005, *t* = −2.81, *p* = 0.005). The average SSI for incorrectly answered melody discrimination items was higher (*M* = 34.7, *SD* = 21.3) than for correctly answered items (*M* = 31.8, *SD* = 20.6). Adjusting for years of musical training and trial length did not substantially change the results (−0.005, *t* = −2.06, *p* = 0.04).

### Effect of Motor Synchronization Condition Compared to Listening Condition

In the next part of our analyses, we investigated whether tapping provided any benefit for MDP compared to listening only. Contrary to our expectation, on average participants performed worse in the tapping condition (*M* = 69.1, *SD* = 10.6) than during the listening only condition (*M* = 71.0, *SD* = 11.0; −1.87 mean difference of tapping and listening, *t* = −2.12, *p* = 0.04).

### Effects of Individual Differences in Musical Training and Brain Anatomy on Melody Discrimination and Tapping Performance

#### Musical Training

Longer musical training was associated with better MDP (Table 2). Among musicians, earlier start of musical training tended to be related to better MDP. However, in our sample starting age was strongly correlated with duration of musical training (*r* [Pearson] = −0.38, *p* < 0.001). When we entered both variables simultaneously in the model, the effect of years of musical training hardly changed but the effect of starting age largely disappeared (Table 2). Furthermore, longer musical training was associated with a better tapping performance (smaller SSI; Table 2). Starting age of musical training showed no significant effect (Table 2).

**TABLE 2:** Effects of years of musical training and starting age of musical training on melody discrimination performance and tapping performance in the total sample.

		**Difference**					**Difference**				
**Model**	**Determinant**	**in MDP [%]**	**95% CI**		***t***	***p***	**in SSI [ms]**	**95% CI**		***t***	***p***
1	Musical training [per year]	0.89	0.59	1.19	5.84	<0.001	–0.88	–1.39	–0.38	–3.44	<0.001
2	SA of musical training [per log(year)]	–4.97	–10.44	0.49	–1.81	0.07	2.80	–5.00	10.61	0.71	0.48
3	Musical training [per year]	0.76	0.30	1.21	3.30	0.001	–	–	–	–	–
	SA of musical training [per log(year)]	–1.47	–7.06	4.11	–0.52	0.60	–	–	–	–	–

*Linear regression models on MDP and SSI were independently tested. MDP, melody discrimination performance; SSI, sensorimotor simultaneity index; SA, starting age.*

#### Brain Anatomy

In the MRI subgroup, a thicker right but not left Heschl’s gyrus was associated with better MDP (Table 3). When we adjusted for duration of musical training, the effect size of right Heschl’s gyrus became slightly smaller and only borderline statistically significant (Table 3). A thicker left and right Heschl’s gyrus were also associated with better tapping performance (Table 3). When additionally controlling for years of musical training, the effect sizes slightly decreased and only the right Heschl’s gyrus remained significantly associated with tapping performance (Table 3). We did not find a significant association of right superior frontal cortical thickness and SSI (Table 3). There were no substantial differences in any of the effects in the right-handed individuals only.

**TABLE 3:** Effects of Heschl’s gyrus cortical thickness on melody discrimination performance and effects of Heschl’s gyrus cortical thickness and right superior frontal cortex on tapping performance in the MRI subsample.

		**Difference**					**Difference**				
**Model**	**Determinant**	**in MDP [%]**	**95% CI**		***t***	***p***	**in SSI [ms]**	**95% CI**		***t***	***p***
1	Right Heschl’s gyrus thickness [per *SD*]	2.44	0.37	4.51	2.35	0.02	–3.65	–7.02	–0.29	–2.17	0.03
2	Right Heschl’s gyrus thickness [per *SD*]	1.93	–0.10	3.96	1.90	0.06	–3.47	–6.92	–0.02	–2.01	0.049
	Musical training [per year]	0.55	0.11	0.99	2.52	0.01	–0.20	–0.94	0.55	–0.53	0.60
3	Left Heschl’s gyrus thickness [per *SD*]	0.57	–1.64	2.77	0.51	0.61	–3.71	–7.15	–0.27	–2.15	0.03
4	Left Heschl’s gyrus thickness [per *SD*]	–0.40	–2.60	1.80	–0.37	0.72	–3.54	–7.18	0.10	–1.94	0.06
	Musical training [per year]	0.66	0.20	1.12	2.85	0.01	–0.11	–0.88	0.65	–0.30	0.77
5	Right SFC thickness [per *SD*]	–	–	–	–	–	–0.53	–4.01	2.94	–0.31	0.76

*Linear regression models on MDP and SSI were independently tested; all models were adjusted for intracranial volume. MDP, melody discrimination performance; SSI, sensorimotor simultaneity index; SD, standard deviation; SFC, superior frontal cortex.*

### *Post hoc* Exploratory Analyses

The unexpected finding of no benefit in motor synchronization condition compared to listening only condition lead us to explore possible moderators of this effect. In additional analyses, we explored whether the net negative effect of tapping compared to listening was present in all individuals, or if we could find characteristics that identified those who showed a benefit. While on group average, the net effect was negative, when we visually explored the pattern of results we found that roughly a third of the participants showed better melody discrimination during tapping than during listening. Two possible factors that we identified and that we were able to explore in the existing dataset were motor synchronization abilities and brain anatomy.

#### Motor Synchronization Abilities

We first explored whether those individuals that showed a beneficial effect of tapping tended to be individuals with good tapping abilities. Individuals with strong motor synchronization abilities were more likely to show an overall beneficial effect (better MDP in the tapping condition) and individuals with weaker motor synchronization abilities an overall harmful effect of tapping (better melody discrimination in the listening condition). However, this effect was not statistically significant (−0.05, *t* = −0.93, *p* = 0.36).

#### Brain Anatomy

**FIGURE 3:**
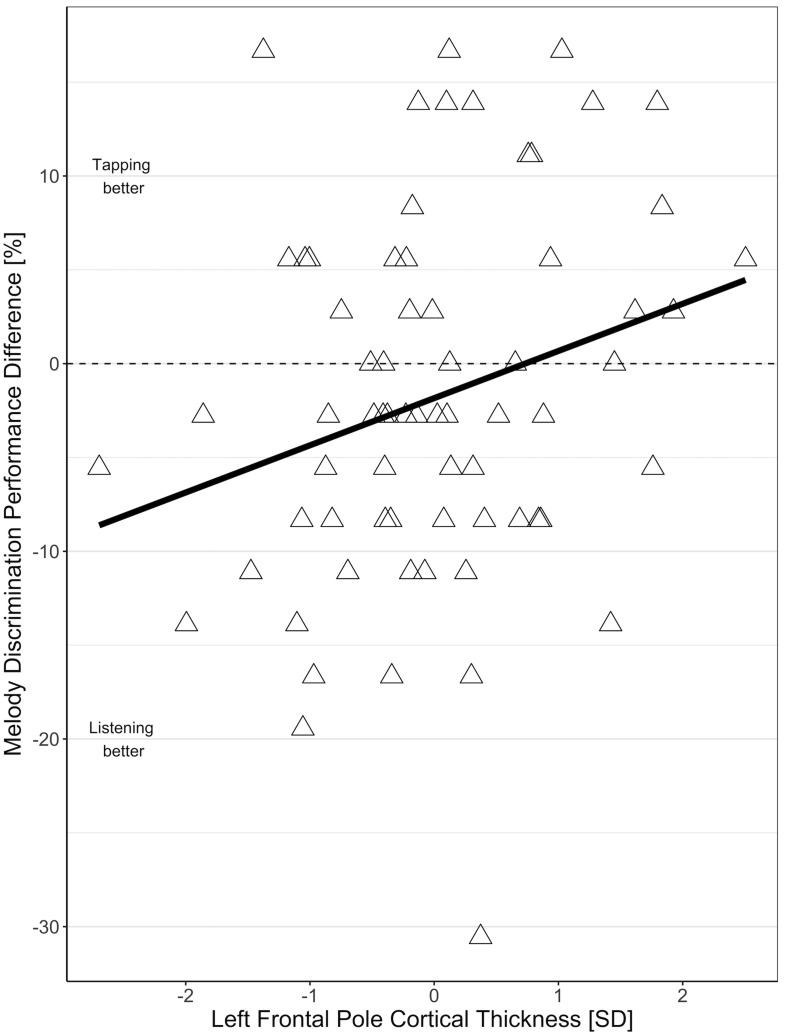
Association of the difference between melody discrimination performance in tapping and listening condition with left frontopolar cortical thickness.

In the MRI subgroup, the effect of condition (tapping versus listening) on MDP was comparable to the effect in the larger behavioral sample (−1.84 mean difference between tapping and listening, *t* = −1.59, *p* = 0.12) i.e., overall performance was worse during tapping than during listening only. This relation depended on frontal pole cortical thickness with individuals with thinner frontal poles showing worse performance during tapping than during listening, and individuals with thicker frontal poles showing better melody discrimination when tapping (Figure 3). The interaction of frontal pole cortical thickness and condition (tapping versus listening) on MDP was statistically significant for the left frontal pole (2.5% increase in the difference between tapping and listening condition per 1 SD increase in frontal pole thickness, *t* = 2.15, *p* = 0.04), but not for the right frontal pole (1.35% increase in the difference between tapping and listening condition per 1 SD increase in frontal pole thickness, *t* = 1.14, *p* = 0.26). We did not find significant effects for any other frontal region that we tested. There were no substantial differences in any of the effects in the right-handed individuals only.

## Discussion

We found a beneficial effect of motor synchronization on auditory discrimination using naturalistic and meaningful auditory material: melodies in the Western tonal system. Although melody discrimination improved with better tapping synchrony, it was worse in the tapping than the listening only condition. Longer musical training and a thicker Heschl’s gyrus were associated with better melody discrimination (auditory component) and with better tapping synchrony (motor component). In our *post hoc* analyses, we found that frontal brain structures modified the association between condition and MDP in that individuals with a thicker left frontopolar cortex performed better when they were required to tap during the melody discrimination task, whereas individuals with a thinner left frontopolar cortex performed better when they were listening only.

We designed an experiment by adding a tapping task to an existing melody discrimination task. Our combined task showed good split-half reliability of our main outcome score MDP. The mean MDP (*M* = 70%) was slightly lower compared to a previous experiment (*M* = 76%) (Foster and Zatorre, 2010b). This could be due to sample differences or because adding the tapping component increased the global difficulty level. However, there were no bottom or ceiling effects according to the distributions of the data. There were no effects of fatigue or of learning in MDP during the course of the experiment. We also could not find effects of trial order on MDP.

The performance scores for auditory-motor synchronization covered a wide range in our sample. Comparisons to other studies that investigated tapping are difficult due to different methods of computing tapping accuracy. However, it seems that tapping accuracy in our sample was on average lower than in other studies (Jäncke et al., 2000; Lehéricy et al., 2006). This could be due to the fact that participants tapped with the non-dominant hand, and that our instruction stressed melody discrimination as the main task.

### Effect of Motor Synchronization on Auditory Processing

Our study was based on a previous study that found a facilitation of auditory perception by top–down motor control of tapping, which was explained by the theory of active sensing (Morillon et al., 2014). We implemented the same experimental conditions of tapping and listening, but used different stimuli and a different auditory task. The stimuli used in the previous study were non-melodic tone sequences that did not follow musical rules, and the judgment required was about average pitch of the tones. Our study used melodies according to Western musical syntax rather than random tone sequences, and the task was more complex, since it required identification of a single tone that was different between the melodies. In our analyses, we excluded effects of short-term training or fatigue, stimulus-specific effects such as length of melody, and musical training. In contrast to the previous study, we did not only analyze differences between tapping and listening conditions, but also analyzed the effect of tapping accuracy on auditory processing. Here, in order to understand the effect of synchronized motor action on MDP, we analyzed the effect of tapping accuracy on melody discrimination both at trial level and subject level. On both levels, variations in the tapping performance are meaningful. At the trial level, the tapping score represents dynamic changes in the tapping performance as a state, which possibly depends on other variables such as situational attention and practice effects. At the subject level, the tapping score represents the overall motor synchronization performance as a trait, which varies across individuals, and might be influenced by the training background of the person.

In line with our hypothesis, we found that tapping improved melody discrimination on a trial-by-trial basis, after controlling for the amount of musical training. Moreover, participants who tapped more precisely performed better on the melody discrimination task, regardless of musical training. The fact that tapping accuracy was related to MDP is consistent with the theory of active sensing. However, we failed to replicate the basic finding of tapping benefit that was found in the previous study (Morillon et al., 2014) when we compared the two conditions. Overall, the performance in the tapping condition was worse than in the listening only condition, and this was an unexpected finding.

A possible explanation is that two different effects are reflected in our data. Our analyses of tapping accuracy can be interpreted as evidence for a benefit of auditory-motor synchronization as predicted by active sensing. Furthermore, in a *post hoc* analysis we found that individuals with strong motor synchronization abilities, the “good tappers,” tended to show a true benefit (better auditory discrimination during tapping than during listening) while the individuals with weaker tapping abilities tended to show the detrimental effect that dominated the overall group average effect. Thus, while tapping can be beneficial, there seems to be another process that counteracts this beneficial effect in our data, which might be dual-task inference. Our task can be conceived as two parallel, and possibly competing tasks. One possibility is a competition for working memory. The melody discrimination task highly depends on working memory in terms of memorizing single notes to detect differences. Previous research also shows that timing processing for repetitive movement requires working memory (Holm et al., 2017). Although the cognitive operations in the motor and auditory domain are different, due to the close connections of the two systems (Zatorre et al., 2007), we consider it possible that the two tasks competed for similar resources in terms of working memory capacity. The impact of the competition for working memory resources might have been stronger here than in the previous study (Morillon et al., 2014), because estimating an average pitch (without regard to sequential order) might require less working memory capacity than comparing two consecutive melodies note by note. Moreover, there are a few other factors that might have rendered our task more difficult, and that might have resulted in a competition for resources in terms of cognitive load. Compared to the task used by Morillon et al. (2014) our reference beat was twice as fast (320 ms versus 667 ms). Besides, most participants tapped with their non-dominant left hand, which increased difficulty and might have reduced cognitive resources available for melody discrimination. Our melodies were shorter than in previous studies and lengths of the melodies varied, adding an element of unpredictability. On any given trial, participants did not know how long the melody would be and how long they would have to synchronize. This might have rendered our tapping task more challenging, which might have increased the dual task effort and might have thus reduced the beneficial effect of tapping compared to the previous findings. We conclude that tapping can impair auditory processing under certain conditions, but that individual and trial-by-trial differences in auditory-motor synchronization can nevertheless counter-act any additional cognitive load imposed on auditory perception by entraining attention.

### Effects of Musical Training and Cortical Thickness on Melody Discrimination

In order to understand the auditory component of the auditory-motor-interaction, we examined the effect of musical training and Heschl’s gyrus cortical thickness on MDP. We replicated previous findings that more musical training is associated with better melody discrimination (Foster and Zatorre, 2010b). In addition, we found that the younger people are when they start with musical training the better their melody discrimination, in line with previous results by Foster and Zatorre (2010b). However, musical training years and starting age where highly correlated in our sample. When we assessed them jointly, only years of musical training remained associated with MDP. Such beneficial effects of musical training could also reflect other underlying factors such as cognitive abilities or general intelligence which has been found to be higher in musicians (Schellenberg, 2011; Silvia et al., 2016) and which is also related to timing accuracy in isochronous tapping (Ullen et al., 2008). We did not have a measure of general intelligence in our study, which limits our ability to conclude on possible confounding through intelligence.

Additionally, we showed an association of right Heschl’s gyrus cortical thickness and MDP, which is in line with previous studies showing the Heschl’s gyrus being relevant for pitch discrimination (Zatorre, 1988; Patterson et al., 2002; Foster and Zatorre, 2010b; Norman-Haignere et al., 2013). When we included musical training in the model, the effect size of right Heschl’s gyrus cortical thickness decreased and was no longer statistically significant. This pattern is consistent with previous observations (Foster and Zatorre, 2010b). Our finding of an effect in the right but not the left Heschl’s gyrus is consistent with a right-hemisphere advantage for pitch discrimination of the Heschl’s gyrus (Zatorre and Belin, 2001).

### Effects of Musical Training and Cortical Thickness on Tapping Performance

We showed a positive effect of musical training on tapping accuracy, with more training showing less deviation from tone onset. This finding is in line with previous studies that showed that musical training can improve rhythmic perception and production (Kincaid et al., 2002; Chen et al., 2008; Repp, 2010). For the starting age of musical training, we did not find a significant association with tapping accuracy.

A thicker Heschl’s gyrus was associated with more precise tapping, also when controlling for years of musical training. This is in line with a previous lesion study showing the Heschl’s gyrus to be necessary for retention and reproduction of a precise analog representation of auditory rhythmic patterns (Penhune et al., 1999). We extend previous research by showing an association of Heschl’s gyrus cortical thickness and rhythmic motor tapping, revealing its role in motor synchronization.

Previous studies report about the importance of the premotor cortex for rhythmic tapping (Kung et al., 2013; Repp and Su, 2013). In our experiment participants were instructed to tap with their left index finger, thus we hypothesized to find an association of the right superior frontal cortical thickness and tapping accuracy, which we did not find. One possible explanation is, that the superior frontal cortex region we extracted using the Desikan atlas (Desikan et al., 2006) is a large brain region which codes for various different brain functions and not solely for (finger) motor control. One way to improve statistical power and specificity of analyses in following studies could be using ROIs based on functional activations during the same task rather than anatomically defined ROIs. An additional limitation of our study is that the image contrast in subcortical regions was not sufficient to evaluate further effects of subcortical regions.

### Moderating Effect of Frontal Pole Cortical Thickness on Melody Discrimination

The finding of the overall detrimental effect of tapping compared to listening in our study and the possible interpretation of dual-task interference prompted us to conduct further analyses to explore a possible moderating role of frontal areas. In our *post hoc* analyses, we found that the effect of condition on melody discrimination was dependent on frontal pole cortical thickness: Individuals with thinner frontal poles showed worse performance during tapping than during listening, while individuals with thicker frontal poles showed better melody discrimination while tapping compared to listening. This fits with previous reports of the involvement of the frontal pole in multi-tasking (Koechlin et al., 1999; Braver and Bongiolatti, 2002; Gilbert et al., 2006). This region is important for coordination, monitoring, and integration of subgoal processes within the working memory (Braver and Bongiolatti, 2002). Those individuals with a thicker frontal pole supposedly have more capacity in a multi-tasking brain region. This might enhance their ability to conduct two tasks at a time with fewer costs, and thus they show improved MDP when additionally tapping. Our results especially fit the argumentation, that the frontal pole is important for keeping in mind one main goal while pursuing other tasks (Koechlin et al., 1999). Our participants had to decide whether two melodies in a row were the same or not, which could be considered the main goal, and meanwhile, in half of the trials, to tap along with the beat. Although we found the direction of effect to be the same for both the left and right frontal poles, the effect size was larger, and only statistically significant, for the left frontal pole. Whether this difference is real or due to our relatively small sample size needs to be assessed in future studies.

Our results support the simultaneous involvement of two different mechanisms – the alignment of movement with sensory input and the alignment of temporal attention – in the effect of motor synchronization on auditory function (Morillon et al., 2014). People can benefit from tapping, when they tap accurately enough to align their motor processing with the auditory input. Additionally, they need to align their attention to benefit from this sensory enhancement. According to our findings Heschl’s gyrus might be important for auditory and motor processing and one of the neural correlates of the attentional process might be the frontal pole. A role of additional structures in the fronto-parietal attention network (Corbetta and Shulman, 2002; Buschman and Miller, 2007) and subcortical motor regions (Zatorre et al., 2007; Kung et al., 2013) is likely and could be explored in future imaging studies, e.g., using functional MRI.

Our findings extend previous findings of a beneficial effect, which supports the notion that such paradigms could be translated to the clinic. In clinical applications, so far, synchronous motor movements have been mainly used to enhance speech production rather than processing, e.g., in melodic intonation therapy for stroke (Norton et al., 2009). It remains to be assessed whether the benefits of auditory-motor training transfer to daily life hearing skills. One next step might be to extend this motor enhancement of auditory processing further to non-musical processing such as discrimination of speech sounds.

## Conclusion

In this study, we found that motor synchronization can exert a beneficial effect on auditory discrimination of complex, meaningful material. However, we also showed that a detrimental effect can occur due to dual-task inference. It will be an important challenge to design interventions that balance task difficulty, stimulus complexity and practical relevance of the material to achieve a real benefit. Besides, we disentangled multiple influences on auditory-motor synchronization. We demonstrated the effects of previous experience in musical training and anatomical variability of relevant brain regions on auditory and motor aspects of task performance. We found that structural brain differences are related to the extent to which an individual can benefit from motor synchronization in a complex listening task. Further studies will have to corroborate these findings. If confirmed, our findings could have important implications for the development of personalized auditory-motor training programs to enhance hearing ability.

## Data Availability Statement

The datasets for this manuscript are not publicly available because of data protection regulations. Requests to access the datasets should be directed to MB, monique.breteler@dzne.de.

## Ethics Statement

The studies involving human participants were reviewed and approved by this study was carried out in accordance with the recommendations of the International Council for Harmonisation (ICH) Good Clinical Practice (GCP) standards (ICH-GCP) with written informed consent from all subjects. The protocol was approved by the ethics committee of the University of Bonn, Medical Faculty. The patients/participants provided their written informed consent to participate in this study.

## Author Contributions

NM and SH conceptualized the research question and study design. NM and SH analyzed the data. JK gave advice on MRI data analyses. All authors interpreted the results. NM drafted the manuscript. MB and SH critically reviewed and significantly contributed to the manuscript. All authors provided critical feedback and helped shape the research, analyses and manuscript. All authors approved the final version of the manuscript for publication.

## Conflict of Interest

The authors declare that the research was conducted in the absence of any commercial or financial relationships that could be construed as a potential conflict of interest.
